# Impact of Wearing Masks, Hand Hygiene, and Social Distancing on Influenza, Enterovirus, and All-Cause Pneumonia During the Coronavirus Pandemic: Retrospective National Epidemiological Surveillance Study

**DOI:** 10.2196/21257

**Published:** 2020-08-20

**Authors:** Nan-Chang Chiu, Hsin Chi, Yu-Lin Tai, Chun-Chih Peng, Cheng-Yin Tseng, Chung-Chu Chen, Boon Fatt Tan, Chien-Yu Lin

**Affiliations:** 1 MacKay Children's Hospital Taipei Taiwan; 2 Department of Medicine, MacKay Medical College New Taipei Taiwan; 3 Hsinchu MacKay Memorial Hospital Hsinchu Taiwan; 4 Teaching Center of Natural Science, Minghsin University of Science and Technology Hsinchu Taiwan; 5 National Taiwan University, Hsinchu Branch Hsinchu Taiwan

**Keywords:** novel coronavirus, COVID-19, SARS-CoV-2, pandemic, influenza, pneumonia, hygiene, social distancing, prevention, incidence, surveillance

## Abstract

**Background:**

The coronavirus disease (COVID-19) pandemic is an important health crisis worldwide. Several strategies were implemented to combat COVID-19, including wearing masks, hand hygiene, and social distancing. The impact of these strategies on COVID-19 and other viral infections remains largely unclear.

**Objective:**

We aim to investigate the impact of implemented infectious control strategies on the incidences of influenza, enterovirus infection, and all-cause pneumonia during the COVID-19 pandemic.

**Methods:**

We utilized the electronic database of the Taiwan National Infectious Disease Statistics System and extracted incidences of COVID-19, influenza virus, enterovirus, and all-cause pneumonia. We compared the incidences of these diseases from week 45 of 2016 to week 21 of 2020 and performed linear regression analyses.

**Results:**

The first case of COVID-19 in Taiwan was reported in late January 2020 (week 4). Infectious control strategies have been promoted since late January. The influenza virus usually peaks in winter and decreases around week 14. However, a significant decrease in influenza was observed after week 6 of 2020. Regression analyses produced the following results: 2017, R^2^=0.037; 2018, R^2^=0.021; 2019, R^2^=0.046; and 2020, R^2^=0.599. A dramatic decrease in all-cause pneumonia was also reported (R^2^ values for 2017-2020 were 0.435, 0.098, 0.352, and 0.82, respectively). Enterovirus had increased by week 18 in 2017-2019, but this was not observed in 2020.

**Conclusions:**

Using this national epidemiological database, we found a significant decrease in cases of influenza, enterovirus, and all-cause pneumonia during the COVID-19 pandemic. Wearing masks, hand hygiene, and social distancing may contribute not only to the prevention of COVID-19 but also to the decline of other respiratory infectious diseases. Further studies are warranted to elucidate the causal relationship.

## Introduction

Coronavirus disease (COVID-19), which is caused by severe acute respiratory syndrome coronavirus 2 (SARS-CoV-2), has engendered a substantial health burden worldwide, although the full impact of this virus remains largely unknown [1,2]. As of May 2020, Taiwan had succeeded in containing COVID-19 without a lockdown, recording just 441 confirmed cases (19.2 cases per 1 million residents) [3]. Many strategies have been implemented since mid-January, such as boundary control, use of masks, hand hygiene, and social distancing [4,5]. Advances in technology also contributed to the control of this novel pandemic, including big data analysis, proactive tests, and a real-time, web-based dashboard to track COVID-19 [4,6]. Big data analytics with smart contact tracing and automated alert messaging for self-restriction were used to effectively contain infected patients [7]. Novel technologies contributed to Taiwan’s COVID-19 response.

Although the full pathophysiology of COVID-19 was not known, droplet and contract transmission were believed to be the major transmission route [2]. Wearing a mask was a simple way to prevent viral transmission and decrease disease spread, but the public attitude toward masks varied across countries [8]. The World Health Organization’s recommendation of mask use also varied from time to time [9]. A recent systematic review and meta-analysis showed a significantly lower risk of viral transmission by maintaining a physical distance of 1 meter or more (pooled adjusted odds ratio [aOR] 0.18) [10]. Mask and eye protection use also resulted in a large reduction in the risk of infection (mask use: aOR 0.15; eye protection: aOR 0.22). Additionally, the use of masks by all residents was a key component to successfully combat COVID-19 and may have reduced fear and anxiety [5,11,12]. Briefly, although these customary strategies may have marginal benefits based on current evidence, experts recommend their use during the COVID-19 pandemic [13]. However, the effectiveness of these strategies on other respiratory infections apart from SARS-CoV-2 remains largely unclear. Jefferson et al [14] investigated the effectiveness of physical interventions to reduce the spread of respiratory viruses in their 2011 study and found that wearing masks and hand hygiene were effective against viral transmission; social distancing was not. Barasheed et al [15] explored uptake and effectiveness of masks during mass gatherings in 2016; they found a pooled protective effectiveness with a relative risk of 0.89, but an extremely wide range in the uptake of masks was reported (0.02%-92.8%) [15]. The effectiveness of these traditional strategies and public compliance was not fully disclosed.

The Taiwan government executed a name-based mask rationing plan since late January, and mask factories were recruited. Masks were re-allocated to the general public to ensure availability for all citizens. Mask use and medical care were believed to be key strategies for successful control in Taiwan [11]. Hand hygiene was also promoted in late January, and a significant increase of Google searches for “washing hands” was observed since January 19, 2020 [16]. Citizens of Taiwan were highly motivated to curb the pandemic, and this led to successful outcomes. High uptake and compliance of these traditional practices of infection control, including wearing masks, hand hygiene, and social distancing, were observed in Taiwan. Furthermore, we also observed a noticeable decrease in the number of cases of influenza infection during the COVID-19 pandemic. Taiwan is located in the northern hemisphere where influenza infection is usually prevalent starting in October and peaks in February. We hypothesized that it may be affected by the strategies in place for COVID-19 control and prevention. Therefore, we conducted this retrospective study to investigate the prevalence of other respiratory viral infections using the national surveillance database.

## Methods

### Study Design and Database

Our study was approved by the ethical committee of MacKay Memorial Hospital (No. 20MMHIS140e). Taiwan Centers for Disease Control (CDC) had a comprehensive surveillance system and epidemiological data regarding communicable diseases, influenza virus, enterovirus, and pneumonia that were available on its website [17]. The Taiwan National Infectious Disease Statistics System is a public and nationwide database that provides real-time epidemiologic information to health care personnel.

All suspected cases of COVID-19 received nucleic acid testing following the standard procedure by World Health Organization. All confirmed cases had to be quarantined in hospital and might be discharged after three consecutive negative tests. A diagnosis of influenza virus, enterovirus, and pneumonia were made by physicians based on clinical manifestations, physical examinations, laboratory tests, and imaging studies. The diagnoses were uploaded to the national health insurance system and surveillance system using International Classification of Diseases, Tenth Revision, Clinical Modification (ICD-10-CM) codes [18].

We extracted epidemiological data related to the influenza virus, enterovirus, all-cause pneumonia, and COVID-19. We then compared the weekly cases from October to May (weeks 45 to 21 of next year) for the 2017-2020 period. We compared the incidences of these diseases during the same period and plotted the trendlines. Furthermore, policies and strategies were obtained from the CDC website to demonstrate the time sequences.

### Statistics

The weekly incidences of reported cases were plotted using Microsoft Office, version 2019 (Microsoft Corp), and SPSS, version 23.0 (IBM Corp). Linear regression analyses were performed and R^2^ values were calculated for each year. The equation of linear trend estimation was presented as *y=*α*x+*ß. A positive α coefficient denoted an increase, and a negative α value indicated a decrease. The value of α reflected the slope of the trendline and the magnitude of effects. R^2^, also known as the coefficient of determination, represented the degree of dispersion between individual data and the regression line. The R^2^ value is always between 0% and 100%, and the higher the R^2^ value, the lower the discrepancies between data. An R^2^ value close to 1 represents a reliable fitted regression line.

## Results

**Figure 1:**
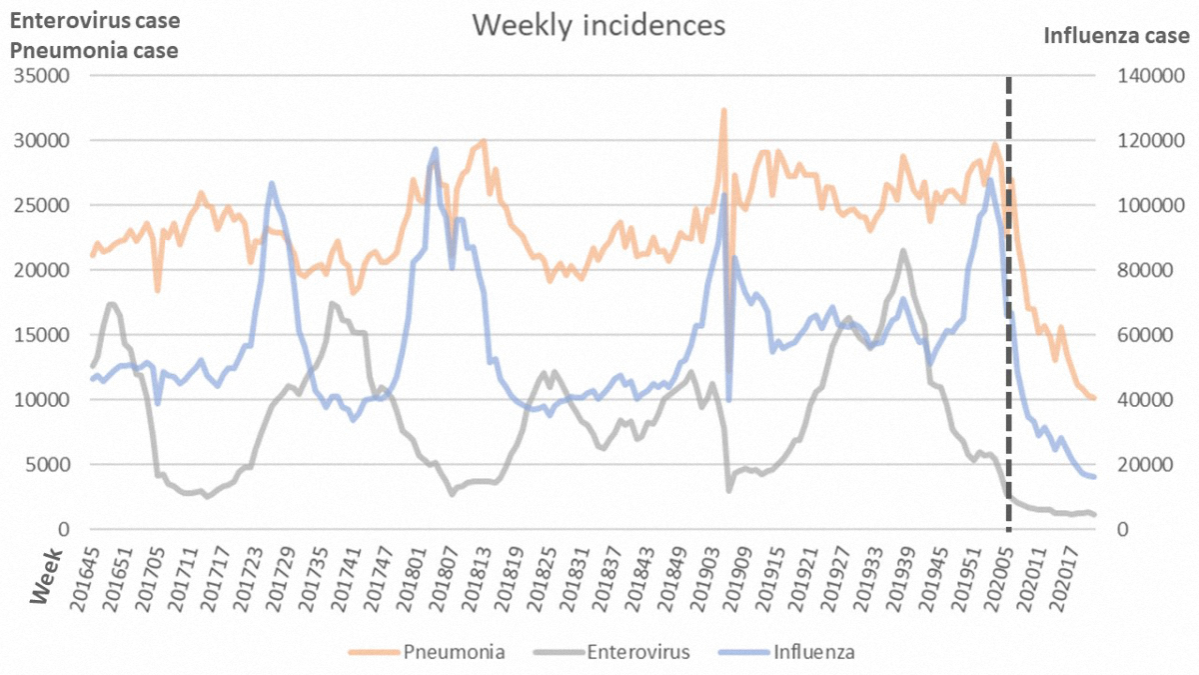
Weekly incidences of influenza, enterovirus, and pneumonia in 2017-2020. Dotted line indicates a significant decrease in all three diseases after week 6 of 2020.

In Taiwan, a unique name-based mask rationing plan was executed, and hand hygiene has been promoted since January 2020 [3]. Social distancing policies recommended a distance of at least 1 meter and 1.5 meters from others in outdoor and indoor settings, respectively, in week 14 of 2020 [3]. We extracted epidemiological data of target diseases from the Taiwan National Infectious Disease Statistics System. As of week 21 of 2020, there were 441 confirmed cases of COVID-19; Taiwan had a relatively controllable situation [2,3]. The incidences of influenza, enterovirus, and pneumonia between week 45 of 2016 and week 21 of 2020 are shown in Figure 1. Seasonality of each disease was observed and a significant decrease in all diseases since week 6 of 2020 were found. The age distribution from week 45 to week 21 of the next year is summarized in Table 1. Fewer patients with influenza, enterovirus, and pneumonia were reported in 2020. Among those with influenza and pneumonia, patients aged 15-24 years had the lowest rates, and approximately one third of patients were younger than 15 years. For patients with enterovirus infection, the majority were younger than 10 years. Compared with 2019, less than half of patients had enterovirus infection in 2020 (106,985 vs 220,865).

**Table 1:** Age distribution of people with respiratory viral infection during the study period (week 45 to week 21 of the next year).

Disease		Year			
		2017	2018	2019	2020
**Influenza, n**		1,405,539	1,960,252	1,838,406	1,518,787
	0-4 years, n (%)	222,203 (15.81)	267,395 (13.64)	269,477 (14.66)	205,911 (13.56)
	5-14 years, n (%)	263,076 (18.72)	418,752 (21.36)	377,639 (20.54)	311,008 (20.48)
	15-24 years, n (%)	114,929 (8.18)	182,663 (9.32)	161,034 (8.76)	129,450 (8.52)
	25-64 years, n (%)	567,302 (40.36)	806,152 (41.12)	759,040 (41.29)	643,141 (42.35)
	≥65 years, n (%)	238,029 (16.94)	285,290 (14.55)	271,216 (14.75)	229,277 (15.1)
**Enterovirus, n**		218,969	171,401	220,865	106,985
	0-2 years, n (%)	69,812 (31.88)	47,332 (27.61)	58,277 (26.39)	30,712 (28.71)
	3-4 years, n (%)	61,824 (28.23)	45,190 (26.37)	64,160 (29.05)	28,397 (26.54)
	5-9 years, n (%)	60,562 (27.66)	51,266 (29.91)	71,069 (32.18)	30,079 (28.12)
	10-14 years, n (%)	11,312 (5.17)	11,913 (6.95)	12,450 (5.64)	5908 (5.52)
	≥15 years, n (%)	15,459 (7.06)	15,700 (9.16)	14,909 (6.75)	11,889 (11.11)
**Pneumonia, n**		668,070	725,014	730,414	593,292
	0-4 years, n (%)	163,835 (24.52)	160,827 (22.18)	161,670 (22.13)	117,323 (19.77)
	5-14 years, n (%)	142,120 (21.27)	148,408 (20.47)	151,033 (20.68)	115,580 (19.48)
	15-24 years, n (%)	28,368 (4.25)	31,242 (4.31)	29,487 (4.04)	25,201 (4.25)
	25-64 years, n (%)	198,359 (29.69)	229,681 (31.68)	233,855 (32.02)	203,163 (34.24)
	≥65 years, n (%)	135,388 (20.27)	154,857 (21.36)	154,369 (21.13)	132,025 (22.25)

**Figure 2:**
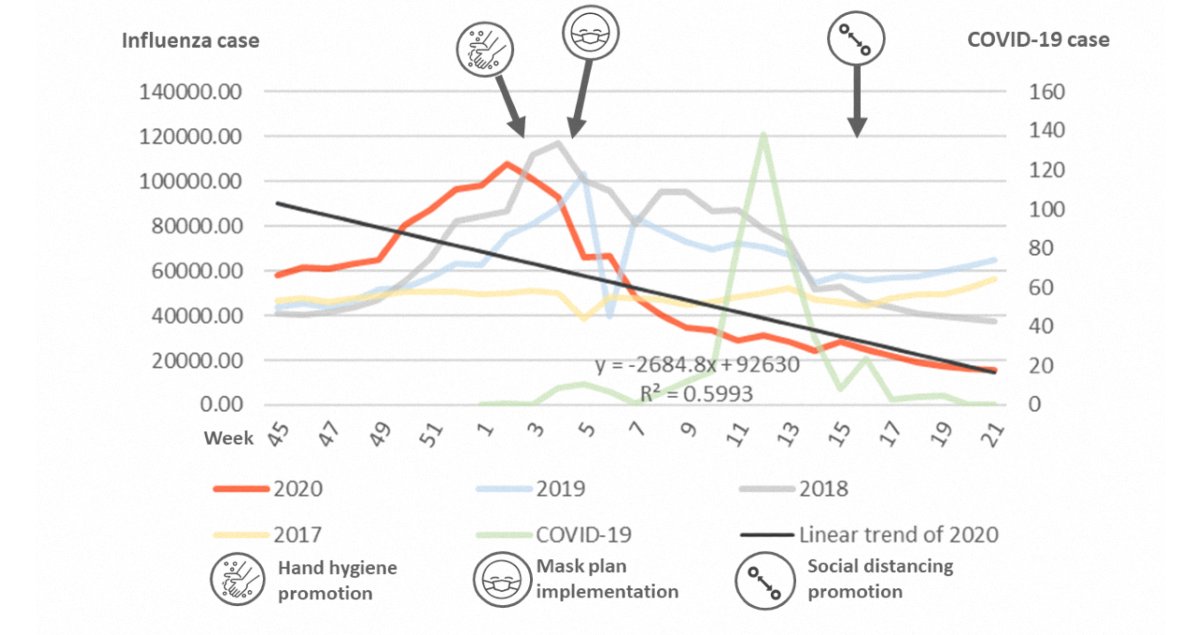
Incidences of influenza in 2017-2020. COVID-19: coronavirus disease.

**Figure 3:**
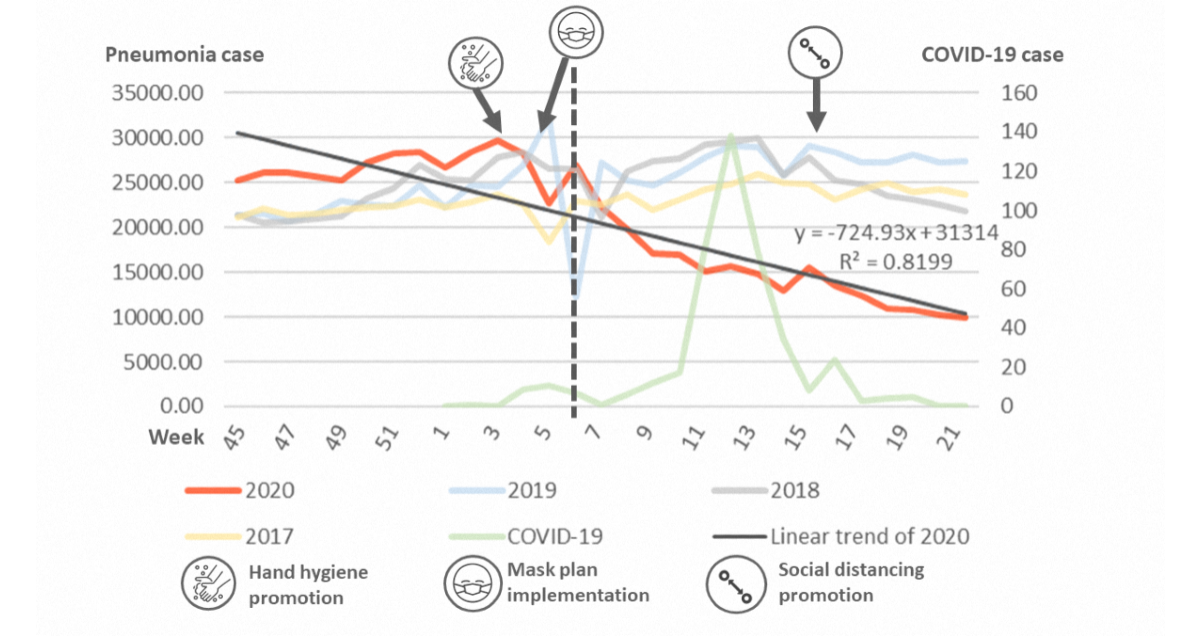
Incidences of all-cause pneumonia in 2017-2020. A dramatic decrease in pneumonia from week 6 of 2020 is shown by the dotted line. COVID-19: coronavirus disease.

**Figure 4:**
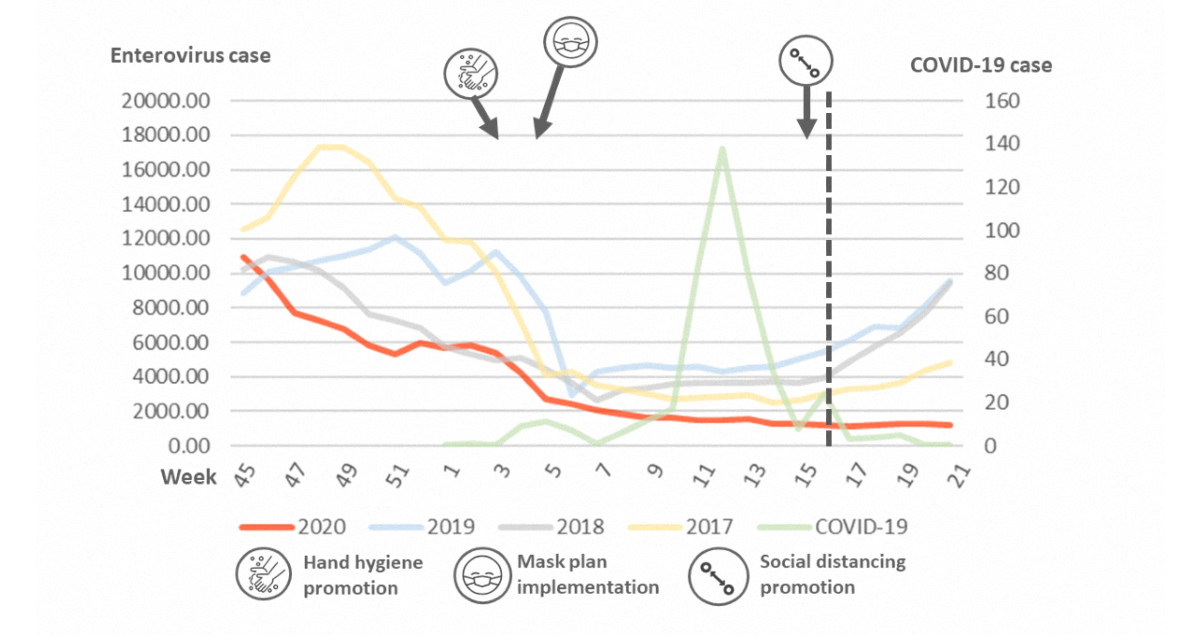
Incidences of enterovirus in 2017-2020. Dotted line marks the increase that is usually seen in enterovirus infection cases in week 16, although this was not observed in 2020. COVID-19: coronavirus disease.

We further plotted the reported cases of influenza from week 45 to week 21 of the next year in Figure 2. There was no significant variation between weeks in 2017, but influenza increased rapidly around week 50 of 2018 and decreased around week 14 of 2019. In 2020, the COVID-19 pandemic began in week 4, during which a dramatic decrease in influenza was observed. The R^2^ values for 2017-2020 were 0.037, 0.021, 0.046, and 0.599, respectively (Table 2). This dramatic decrease was more significant in all-cause pneumonia from week 6 onward (Figure 3). Enterovirus infection was common in Taiwan and usually increased by week 16 (Figure 4), although no such increase occurred in 2020.

**Table 2:** Regression analyses results (R^2^) for 2017-2020.

Disease	Year			
	2017	2018	2019	2020
Influenza	0.037	0.021	0.046	0.599
Enterovirus	0.72	0.256	0.359	0.834
All-cause pneumonia	0.435	0.098	0.352	0.82

## Discussion

Utilizing the national electronic epidemiologic database, we found a concomitant decrease in influenza, enterovirus, and all-cause pneumonia during the COVID-19 pandemic. Infectious control measures, including wearing masks, hand hygiene, and social distancing, may contribute not only to the prevention of COVID-19 but also to decreases in the incidence of other viral infections and pneumonia.

Advances in technology also contributed to COVID-19 response. Several novel techniques had been applied to control COVID-19, including an interactive web-based dashboard, big data analysis, mobile technology, and social media platforms. Internet-based digital citizen science is the crucial component for tackling pandemics in the 21th century [19-23]. Our study utilized electronic epidemiologic statistics and provided timely preliminary findings. A combination of novel technology and traditional infectious control measurements played crucial roles to fight the pandemic. Controversies regarding the effectiveness of traditional strategies exist and the uptake of these strategies varied across countries. Although these strategies are straightforward for reducing respiratory viral transmission, convincing evidence supporting their effectiveness is lacking. Most studies were observational studies or simulation models, and a strong recommendation was not achieved [24]. For COVID-19, asymptomatic patients may spread disease so universal masking in communities was recommended in some areas [8,12,13,25]. However, the effectiveness of masks is doubted; different fitted filtration efficiencies were observed in different masks [26]. Furthermore, wearing masks, especially N95 respirators, is uncomfortable, which can contribute to noncompliance. Generally speaking, uptake of masks was widely accepted in Asian countries, and high compliance of mask use was observed during the pandemic [8,27]. Additionally, a shortage of masks was an important issue, and Taiwan implemented a name-based mask distribution system and rationing plan to ensure availability of masks for purchase [5,28]. Although a direct comparison with randomized controlled trials was not available, our study found a relatively controllable COVID-19 situation in Taiwan. As of the 21st week of 2020, there were 441 confirmed cases of COVID-19 in Taiwan. These traditional strategies were effective for prevention and control.

We found these strategies were effective not only in reducing COVID-19 but also other respiratory viruses. However, it is difficult to investigate the independent and combined effects of each strategy. Although wearing masks, hand hygiene, and social distancing are straightforward, it is difficult, and may be unethical, to conduct a randomized controlled trial to compare their protective effects during the pandemic. In Taiwan, policies for wearing masks and hand hygiene were implemented in late January and social distancing was promoted in week 14. The observed decrease began in week 6, and success may be mainly attributed to wearing a mask and hand hygiene. Moreover, previous studies found social distancing had not significantly reduced transmission of influenza and other viruses [14,29,30]. SARS-CoV-2 is highly contagious; the effects of these practices may vary in areas with different viruses, societies, cultures, health care resources, population densities, disease prevalence, and proportion of subclinical carriers. It is a challenge to determine the effectiveness of these practices on outcomes since the results are drawn from descriptive analyses. There were many factors such as comorbidity, health care access during COVID-19, age, sex, time of the year, mask wearing behaviors (eg, all the time, sometimes), hand washing frequencies, etc, that may affect incidence rates. Nevertheless, the entire causal relationship between infectious control measurement and viral transmission was not easily clarified. Further studies are warranted to investigate the independent and combined effects of these practices.

Our study demonstrated a significant decrease in respiratory viral diseases after the implementation of these practices using linear trend estimation. Time-series analysis was also a useful tool to predict the trendline based on previous epidemiological data [31]. However, COVID-19 is a novel situation; thus, a precise prospective prediction may be not feasible at present. Additionally, seasonality is an important factor for infectious diseases, and the onset of the pandemic occurred approximately 6 months ago. The impact of seasonality on COVID-19 remains unclear. Therefore, we decided to compare the incidences of the same period (week 45 to week 21 of the next year) in different years and plot the trendlines using linear regression analyses [32,33], which is more meaningful and intuitive. However, the COVID-19 situation changes rapidly and the optimal strategies to combat COVID-19 have also changed rapidly. Timely and continuous surveillance and international cooperation are crucial for successful epidemiological studies.

Since the onset of the COVID-19 pandemic, aggressive infection-control measures were implemented, and a decrease in the occurrence of other infectious diseases was observed. The reason for the observed decrease may be multifactorial, with other factors such as virus competition contributing as well [34-36]. Although coinfection with 2 or more viruses were not uncommon, competition for resources between viruses was observed [35,37]. Competition of different subtypes of the influenza A virus was reported in previous study [36]. The observed decreases of other respiratory infections may be the result of competition by SARS-CoV-2. However, there were relatively few cases of COVID-19 in Taiwan. We believe that mask use, hand hygiene, and social distancing controlled the spread of COVID-19 as well as influenza, enterovirus, and all-cause pneumonia. Further studies are warranted to clarify the causal relationship and elucidate the complex interactions between people, viruses, and the environment.

The strength of our study was the use of a national, real-time database on a large population. Our study has some limitations. First, our study was a retrospective epidemiological study. Therefore, the underpinning mechanisms and causal relationships cannot be established; further studies are required for this. Second, the people of Taiwan were strongly motivated to control and prevent infection spread; hence, the independent effects of every single strategy are not easy to confirm. Although hand hygiene and mask use were implemented in the early phase of the pandemic response and seemed to be responsible for the successful decrease in viral transmission, more data on the efficacy of individual strategies is required. Finally, health care resources, accessibility of network, availability of masks, and attitudes toward mask use varied across different societies and countries. The impact of COVID-19 was also different in different areas. Further studies investigating the prevalence of other respiratory viruses in different countries contribute to further understanding the entire impact of COVID-19 and infectious control measurements.

In conclusion, our nationwide epidemiologic study found a significant decrease in influenza, enterovirus, and all-cause pneumonia during the COVID-19 pandemic. Wearing a mask, hand hygiene, and social distancing not only reduced the impact of COVID-19; these strategies also led to a decline in other respiratory infections. Further studies are warranted to clarify this causal relationship.

## Abbreviations

aOR: adjusted odds ratio
CDC: Taiwan Centers for Disease Control
COVID-19: coronavirus disease
ICD-10-CM: International Classification of Diseases, Tenth Revision, Clinical Modification
SARS-CoV-2: severe acute respiratory syndrome coronavirus 2

## References

[1] Easom N, Moss P, Barlow G, Samson A, Taynton T, Adams K, Ivan M, Burns P, Gajee K, Eastick K, Lillie PJ (2020). Sixty-eight consecutive patients assessed for COVID-19 infection: Experience from a UK Regional infectious diseases Unit. Influenza Other Respir Viruses.

[2] (2020). Coronavirus disease (COVID-19) outbreak situation. World Health Organization.

[3] (2020). Coronavirus disease 2019 (COVID-19). Centers for Disease Control and Prevention.

[4] Wang CJ, Ng CY, Brook RH (2020). Response to COVID-19 in Taiwan: Big Data Analytics, New Technology, and Proactive Testing. JAMA.

[5] Chang C, Tan T, Ho T, Chen C, Su T, Lin C (2020). COVID-19: Taiwan's epidemiological characteristics and public and hospital responses. PeerJ.

[6] Dong E, Du H, Gardner L (2020). An interactive web-based dashboard to track COVID-19 in real time. The Lancet Infectious Diseases.

[7] Chen C, Jyan H, Chien S, Jen H, Hsu C, Lee P, Lee C, Yang Y, Chen M, Chen L, Chen H, Chan C (2020). Containing COVID-19 Among 627,386 Persons in Contact With the Diamond Princess Cruise Ship Passengers Who Disembarked in Taiwan: Big Data Analytics. J Med Internet Res.

[8] Feng S, Shen C, Xia N, Song W, Fan M, Cowling BJ (2020). Rational use of face masks in the COVID-19 pandemic. The Lancet Respiratory Medicine.

[9] (2020). Advice on the use of masks in the community, during home care and in health care settings in the context of the novel coronavirus (2019-nCoV) outbreak: interim guidance. World Health Organization.

[10] Chu DK, Akl EA, Duda S, Solo K, Yaacoub S, Schünemann Holger J, COVID-19 Systematic Urgent Review Group Effort (SURGE) study authors (2020). Physical distancing, face masks, and eye protection to prevent person-to-person transmission of SARS-CoV-2 and COVID-19: a systematic review and meta-analysis. Lancet.

[11] Su Vincent Yi-Fong, Yen Y, Yang K, Su W, Chou K, Chen Y, Perng Diahn-Warng (2020). Masks and medical care: Two keys to Taiwan's success in preventing COVID-19 spread. Travel Med Infect Dis.

[12] Klompas M, Morris CA, Sinclair J, Pearson M, Shenoy ES (2020). Universal Masking in Hospitals in the Covid-19 Era. N Engl J Med.

[13] Greenhalgh T, Schmid MB, Czypionka T, Bassler D, Gruer L (2020). Face masks for the public during the covid-19 crisis. BMJ.

[14] Jefferson T, Del Mar CB, Dooley L, Ferroni E, Al-Ansary LA, Bawazeer GA, van Driel ML, Nair S, Jones MA, Thorning S, Conly JM (2011). Physical interventions to interrupt or reduce the spread of respiratory viruses. Cochrane Database Syst Rev.

[15] Barasheed O, Alfelali M, Mushta S, Bokhary H, Alshehri J, Attar A, Booy Robert, Rashid Harunor (2016). Uptake and effectiveness of facemask against respiratory infections at mass gatherings: a systematic review. Int J Infect Dis.

[16] Lin Y, Liu C, Chiu Y (2020). Google searches for the keywords of "wash hands" predict the speed of national spread of COVID-19 outbreak among 21 countries. Brain Behav Immun.

[17] (2020). Taiwan National Infectious Disease Statistics System. Centers for Disease Control.

[18] Lin C, Liu J, Wu C, Hsu R, Hsu W (2020). Decreased Risk of Renal Calculi in Patients Receiving Androgen Deprivation Therapy for Prostate Cancer. Int J Environ Res Public Health.

[19] Ahmad AR, Murad HR (2020). The Impact of Social Media on Panic During the COVID-19 Pandemic in Iraqi Kurdistan: Online Questionnaire Study. J Med Internet Res.

[20] Geldsetzer P (2020). Use of Rapid Online Surveys to Assess People's Perceptions During Infectious Disease Outbreaks: A Cross-sectional Survey on COVID-19. J Med Internet Res.

[21] Huang Y, Wu Q, Wang P, Xu Y, Wang L, Zhao Y, Yao D, Xu Y, Lv Q, Xu S (2020). Measures Undertaken in China to Avoid COVID-19 Infection: Internet-Based, Cross-Sectional Survey Study. J Med Internet Res.

[22] Katapally TR (2020). A Global Digital Citizen Science Policy to Tackle Pandemics Like COVID-19. J Med Internet Res.

[23] Wang P, Lu W, Ko N, Chen Y, Li D, Chang Y, Yen C (2020). COVID-19-Related Information Sources and the Relationship With Confidence in People Coping with COVID-19: Facebook Survey Study in Taiwan. J Med Internet Res.

[24] Nussbaumer-Streit B, Mayr V, Dobrescu AI, Chapman A, Persad E, Klerings I, Wagner G, Siebert U, Christof C, Zachariah C, Gartlehner G (2020). Quarantine alone or in combination with other public health measures to control COVID-19: a rapid review. Cochrane Database Syst Rev.

[25] Cheng VC, Wong S, Chuang VW, So SY, Chen JH, Sridhar S, To KK, Chan JF, Hung IF, Ho P, Yuen K (2020). The role of community-wide wearing of face mask for control of coronavirus disease 2019 (COVID-19) epidemic due to SARS-CoV-2. J Infect.

[26] Sickbert-Bennett Emily E, Samet James M, Clapp Phillip W, Chen Hao, Berntsen Jon, Zeman Kirby L, Tong Haiyan, Weber David J, Bennett William D (2020). Filtration Efficiency of Hospital Face Mask Alternatives Available for Use During the COVID-19 Pandemic. JAMA Intern Med.

[27] (2020). Universal mask-wearing is the most overlooked COVID-19 lifesaver. maskssavelives.org.

[28] Wu Huai-Liang, Huang Jian, Zhang Casper J P, He Zonglin, Ming Wai-Kit (2020). Facemask shortage and the novel coronavirus disease (COVID-19) outbreak: Reflections on public health measures. EClinicalMedicine.

[29] Ahmed F, Zviedrite N, Uzicanin A (2018). Effectiveness of workplace social distancing measures in reducing influenza transmission: a systematic review. BMC Public Health.

[30] Yu D, Lin Q, Chiu AP, He D (2017). Effects of reactive social distancing on the 1918 influenza pandemic. PLoS One.

[31] Dayer MJ, Jones S, Prendergast B, Baddour LM, Lockhart PB, Thornhill MH (2015). Incidence of infective endocarditis in England, 2000–13: a secular trend, interrupted time-series analysis. The Lancet.

[32] Marill KA (2004). Advanced statistics: linear regression, part I: simple linear regression. Acad Emerg Med.

[33] Kraemer MUG, Yang C, Gutierrez B, Wu C, Klein B, Pigott DM, du Plessis Louis, Faria Nuno R, Li Ruoran, Hanage William P, Brownstein John S, Layan Maylis, Vespignani Alessandro, Tian Huaiyu, Dye Christopher, Pybus Oliver G, Scarpino Samuel V, Open COVID-19 Data Working Group (2020). The effect of human mobility and control measures on the COVID-19 epidemic in China. Science.

[34] Pinky L, Dobrovolny HM (2016). Coinfections of the Respiratory Tract: Viral Competition for Resources. PLoS One.

[35] Trinh JT, Zeng L (2017). Virus interactions: cooperation or competition?. Future Microbiol.

[36] Latorre-Margalef N, Brown JD, Fojtik A, Poulson RL, Carter D, Franca M, Stallknecht DE (2017). Competition between influenza A virus subtypes through heterosubtypic immunity modulates re-infection and antibody dynamics in the mallard duck. PLoS Pathog.

[37] Lin C, Hwang D, Chiu N, Weng L, Liu H, Mu J, Liu C, Chi H (2020). Increased Detection of Viruses in Children with Respiratory Tract Infection Using PCR. Int J Environ Res Public Health.

